# *In Vitro* Cestocidal Activity of Thymol on *Mesocestoides corti* Tetrathyridia and Adult Worms

**DOI:** 10.1155/2014/268135

**Published:** 2014-09-03

**Authors:** M. Maggiore, M. C. Elissondo

**Affiliations:** ^1^Laboratorio de Zoonosis Parasitarias, Departamento de Biología, Facultad de Ciencias Exactas y Naturales, Universidad Nacional de Mar del Plata (UNMdP), 3350 Funes Street, 7600 Mar del Plata, Argentina; ^2^Consejo Nacional de Investigaciones Científicas y Técnicas (CONICET), Buenos Aires, Argentina

## Abstract

Nothing is known about the possible effect of thymol or other compounds of essential oils against the adult worms of cestodes. The aim of the present work was to determine *in vitro* cestodicidal activity of thymol against *Mesocestoides corti* adult worms. Moreover, the *in vitro* effect on tetrathyridia was also demonstrated. Tetrathyridia exposed to different concentrations of thymol showed a concentration and time-dependent effect. At lower concentrations, the main change observed was mainly in morphology, with larvae exhibiting an elongation of the body. When tetrathyridia were exposed to higher concentrations, increased surface alterations and damage were detected. The body appeared elongated and flattened, and a complete loss of morphology and microtriches was observed. Thymol was able to kill *M. corti* tetrathyridia, since following inoculation of treated parasites in mice no parasites could be recovered. The effect on *M. corti* adult worms was dose and time-dependent. Changes in motility coincide with the tissue damage were observed at the structural and ultrastructural level. Thymol caused severe damages to both developmental stages analyzed. Damages were more significant in fully segmented worms. The data reported in this paper demonstrate a clear *in vitro* effect of thymol against *M. corti* tetrathyridia and adult worms.

## 1. Introduction

Helminth parasitism remains an underappreciated scourge for humans in most of the developing world. As many as two billion individuals harbor these parasites, all of which often result in chronic debilitating morbidity [1]. Despite this, there are still several unresolved issues in anthelmintic pharmacology for helminthiases of humans. After decades of clinical experience with anthelmintics for the treatment of human infections, most currently used drugs have very poor cestocidal activity. Furthermore, there is a general lack of knowledge about anthelmintic effects upon different developmental stages of cestode parasites, especially due to difficulties in dealing with sexually maturing stages from species infective to humans [2].

*Mesocestoides corti* tetrathyridia have been commonly used for the evaluation of anthelmintic effects [2], but the establishment of an inducible* in vitro* strobilation system [3] now allowed the study of the differential drug susceptibility of distinct developmental forms. A reduced number of compounds have been investigated, using* in vitro* cultured parasites and/or applying* in vivo* rodent models. Tested compounds against tetrathyridia include anti-infective agents like praziquantel and albendazole [4–7]. On the other hand, the effects of praziquantel and albendazole were also evaluated against the adult forms [2].

The control of helminthiases and, generally, of all parasitic diseases is usually made with synthetic anthelmintics. The appearance of resistance stimulated the research of alternatives, such as medicinal plants [8]. Many drugs originate from herbal sources: a century ago, most of the effective drugs were plant based. The development of drugs from plants continues, with drug companies engaged in pharmacological screening of herbs [9]. The pharmaceutical properties of aromatic plants are partially attributed to essential oils. To date, essential oils are presented as valuable therapeutic options against a number of diseases [10]. Moreover, several essential oils and their constituents have been found to possess anthelmintic activity [11, 12].

Recent studies demonstrating the* in vitro* efficacy of several essential oils against* Echinococcus granulosus* protoscoleces implied that these substances and/or their main compounds could also be promising sources of new drugs and may lead to the improvement of natural therapeutic options for the human treatment of cystic echinococcosis [13–15]. The* in vitro* protoscolicidal effect of thymol was demonstrated [13]. Moreover, the* in vitro* and* in vivo* effect of thymol against hydatid cysts was observed (unpublished data). Nevertheless, nothing is known about the possible effect of thymol or other compounds of essential oils against the adult worms.

Thymol is one of the major components of the essential oils of* Thymus* spp. and is a widely known antimicrobial agent. From the analysis of this chemical structure, it could be inferred that, from a biophysical point of view, this compound would have an amphipathic and/or a hydrophobic behavior. This suggests an ability of thymol to partition in the membrane from an aqueous phase as well as a capacity to affect the membrane organization and the surface electrostatics. This assumption may explain the effects of thymol on the permeability of membranes and on the activity of membrane intrinsic proteins such as ATPases or membrane receptors [16].

The aim of the present work was to determine* in vitro* cestodicidal activity of thymol against* Mesocestoides corti* adult worms. Moreover, the* in vitro* effect on tetrathyridia was also demonstrated.

## 2. Materials and Methods

### 2.1. Experimental Animals and Source of Parasites

Animal procedures and management protocols were carried out in accordance with the 2011 revised form of the Guide for the Care and Use of Laboratory Animals published by the U.S. National Institutes of Health. Unnecessary animal suffering was avoided throughout the study. Tetrathyridia initially provided by Dr. Henrique Ferreira (Universidade Federal do Rio Grande do Sul, Brazil) were maintained by serial passages in females of both CF-1 mice and Wistar rats. The animals were inoculated by intraperitoneal injection of 200 mL of larvae (approximately 500 tetrathyridia) in mice and 500 mL of larvae (approximately 1,200 tetrathyridia) in rats, suspended in RPMI 1640 medium modified with HEPES (Emeve Media, 2.05 mM L-glutamine and 25 mM HEPES). After a period of 3–5 months, larvae were harvested from rats and transferred to mice as described by Markoski et al. [3].

### 2.2. Collection of Tetrathyridia

After 3–5 months, the inoculated experimental hosts were euthanized, necropsy was carried out immediately thereafter, and larvae were collected. Yields per infected animal in volumes of 1–9 mL for mice and 1-2 mL for rats were obtained. After harvesting, tetrathyridia were washed 6 times in PBS (with addition of 100 *μ*g/mL streptomycin, 60 *μ*g/mL penicillin, and 50 *μ*g/mL gentamicin) and stored at 4°C in the same antibiotic-added medium for a maximum of 48 hours.

### 2.3. Drug Treatment on Tetrathyridia

Tetrathyridia of* M. corti* were isolated under aseptic conditions from CF-1 mice. Tetrathyridia were cultured in RMPI 1640 medium, supplemented with 100 *μ*g/mL streptomycin, 60 *μ*g/mL penicillin, and 50 *μ*g/mL gentamicin. Cultures were performed on 24 well plates (20 *μ*L of tetrathyridia per well), supplied with 3 mL/well of RPMI 1640 medium, and incubated at 37°C. Thymol (Sigma) was dissolved in dimethyl sulfoxide (DMSO) at a drug concentration of 100 mg/mL and added to the medium resulting in final concentrations of 250, 200, 150, 100, 50, 25, and 10 *μ*g/mL. Tetrathyridia incubated with culture medium alone and with culture medium containing DMSO were used as controls. Exposure to each drug concentration was carried out in quadruplicate. Samples of tetrathyridia for scanning electron microscopy (SEM) were taken after 1 h and 18–20 h (overnight) following incubation.

### 2.4. Determination of Infectivity to Mice

Tetrathyridia (500 per Leighton tube) were cultured in RPMI 1640 medium, containing 60 *μ*g/mL penicillin, 100 *μ*g/mL streptomycin, and 50 *μ*g/mL gentamicin. Cultures were performed in 10 mL of incubation medium at 37°C. Thymol was added to the medium resulting in a final concentration of 250 *μ*g/mL. Tetrathyridia incubated with culture medium containing DMSO were used as controls. Parasites were recovered after 18–20 h (overnight incubation), washed, and used to infect 8 mice by intraperitoneal inoculation (200 *μ*L of larvae per animal, 4 control and 4 treated mice). Animals were housed in a temperature-controlled (22°C ± 1°C), light-cycled (12 h light/dark cycle) room. Food and water were provided ad libitum. After 2 months following infection, mice were euthanized and parasites were recovered from their peritoneal cavity. The efficacy of chemotherapy was estimated through the percentage: (mean from control group-mean from treated group)/mean from control group × 100 (where mean refers to the volume of recovered parasites).

### 2.5. Segmentation Induction

Tetrathyridia segmentation was induced as previously described by Markoski et al. [3]. Briefly, starved cultured larvae were incubated in RPMI 1640 medium containing 0.662% (w/v) trypsin (Gibco) during 24 hours. After induction, cultures were transferred to 24 well plates (20 *μ*L of tetrathyridia per well), supplied with 3 mL/well of RPMI 1640 medium, supplemented with 20% fetal bovine serum (Gibco), and maintained at 39°C for up to 10–12 days. The medium was changed every 2 days to avoid excessive acidification.

### 2.6. Drug Treatment on Adult Worms

Cultured worms, after strobilation induction at 12 days, were submitted to thymol treatment. Cultures were performed on 24 well plates (20 *μ*L of parasites per well), supplied with 3 mL/well of RPMI 1640 medium, and incubated at 37°C without changes of medium. Thymol was dissolved in DMSO and added to the medium resulting in final concentrations of 250, 200, and 150 *μ*L/mL. Worms incubated with culture medium containing DMSO were used as controls. Exposure to each drug concentration was carried out in quadruplicate. Culture plates were observed microscopically during the first hour and changes were photographed. Samples of tetrathyridia for SEM were taken after 15, 30, and 60 min and 18–20 h (overnight) following incubation.

### 2.7. Scanning Electron Microscope

Samples of tetrathyridia and adult worms were processed for SEM as described by Elissondo et al. [17] for* E. granulosus* samples. The fixation time was modified. Briefly, samples were fixed with 3% glutaraldehyde in sodium cacodylate buffer for 48 h at 4°C. Then several washes in cacodylate buffer were made and the specimens were dehydrated by sequential incubations in increasing concentrations of ethanol (50–100%) and were finally immersed in hexamethyldisilazane for 5 min, 1 h, and then overnight. They were then sputter-coated with gold (100 Å thick) and inspected on a JEOL JSM-6460 LV scanning electron microscope operating at 15 kV.

## 3. Results

### 3.1. Drug Treatment on Tetrathyridia

**Figure 1:**
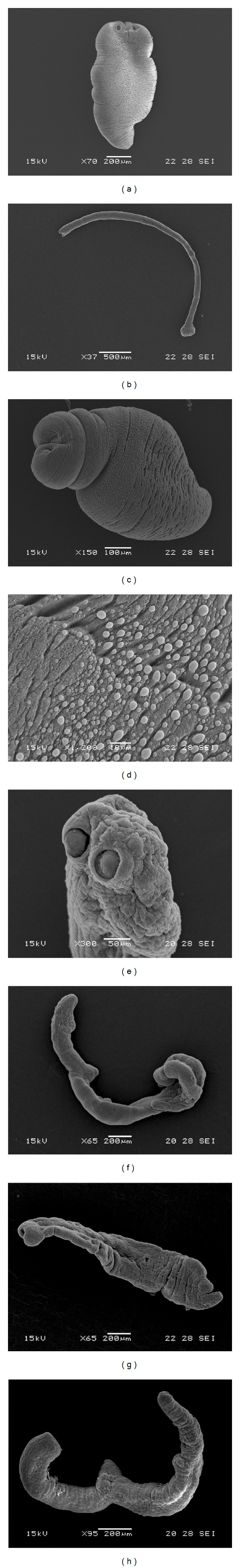
Scanning electron microscopy of* Mesocestoides corti* tetrathyridia incubated* in vitro* with thymol. (a) Control tetrathyridium (70x). (b) Altered tetrathyridium after 1 h following incubation (100 *μ*g/mL). Note the elongation of the body (37x). (c) Thymol-treated tetrathyridium. Observe the blebs and depressions (1 h, 25 *μ*g/mL, 150x). (d) Detail of blebs and depressions (1 h, 25 *μ*g/mL, 150x). (e) Alterations in the tegument of the suckers region (1 h, 150 *μ*g/mL, 300x). (f) Larvae showing the surface damage produced by thymol (1 h, 200 *μ*g/mL, 65x). (g) Altered tetrathyridium (1 h, 250 *μ*g/mL, 65x). The body appeared elongated and flattened. (h) Completely altered tetrathyridium after overnight incubation (250 *μ*g/mL, 95x).

Control tetrathyridia incubated in RPMI medium or in RPMI + DMSO medium remained unaltered, and no changes in ultrastructure were observed (Figure 1(a)). The main change observed after exposure of tetrathyridia to 100, 50, 25, and 10 *μ*g/mL of thymol was mainly in morphology, with larvae exhibiting an elongation of the body (Figure 1(b)). Additionally, the presence of blebs and holes or depressions could be observed (Figures 1(c) and 1(d)). Microtriches remained unaltered after 18–20 h (overnight) of incubation. Increasing the concentration of the drug did not result in a proportional increase in the observable damage.

On the other hand, when tetrathyridia were exposed to 250, 200, and 150 *μ*g/mL of thymol, there were increased surface alterations and damage to the larvae. Tetrathyridia lost their microtriches, the tegument was markedly altered, and the body appeared elongated and flattened (Figures 1(e)–1(g)). Moreover, a decrease in activity was observed. After overnight exposure, complete loss of morphology and paralysis were observed (Figure 1(h)).

### 3.2. Infectivity to Mice

**Figure 2:**
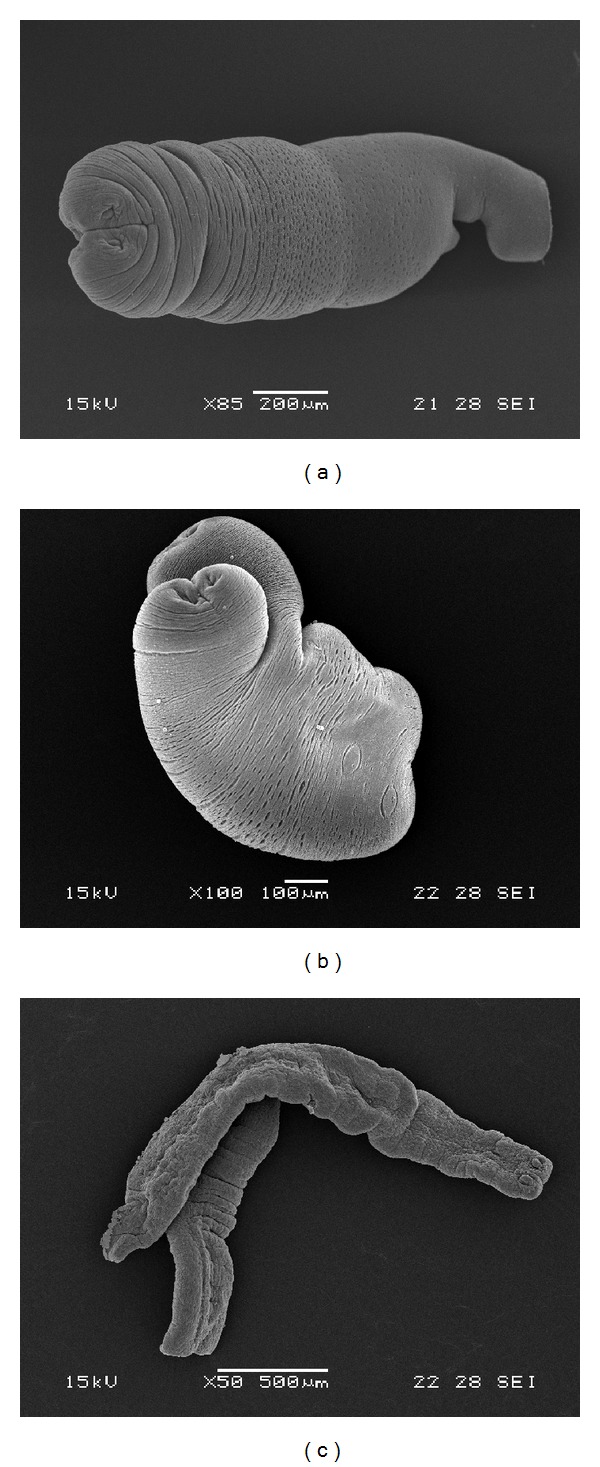
Determination of infectivity to mice. (a) Control tetrathyridium before being injected into mouse (85x). (b) Control tetrathyridium extracted from a mouse after 2 months following infection (100x). (c) Tetrathyridium exposed to thymol (250 *μ*L/mL, overnight) before being injected into mouse. Note the loss of morphology and microtriches. The body appeared elongated and flattened (50x).

Mice were infected with* M. corti* tetrathyridia that had been exposed to thymol (250 *μ*g/mL) for 18–20 h. Control mice were inoculated with untreated tetrathyridia. SEM studies, realized before the infection, demonstrated the unaltered structure of control larvae and the drug-induced ultrastructural damage on treated parasites (Figures 2(a) and 2(c)). After 2 months following infection, mice were necropsied and larvae were collected. The volume of parasites recovered from control animals was 1.68 mL. SEM demonstrated the unaltered appearance of tetrathyridia (Figure 2(b)). On the other hand, no larvae were found in mice infected with thymol-treated tetrathyridia. The efficacy of chemotherapy was 100%. The results from this trial proved the lack of viability of tetrathyridia exposed to thymol (250 *μ*g/mL, overnight), since all of larvae failed to survive following their inoculation into mice.

### 3.3. Drug Treatment on Adult Worms

**Figure 3:**
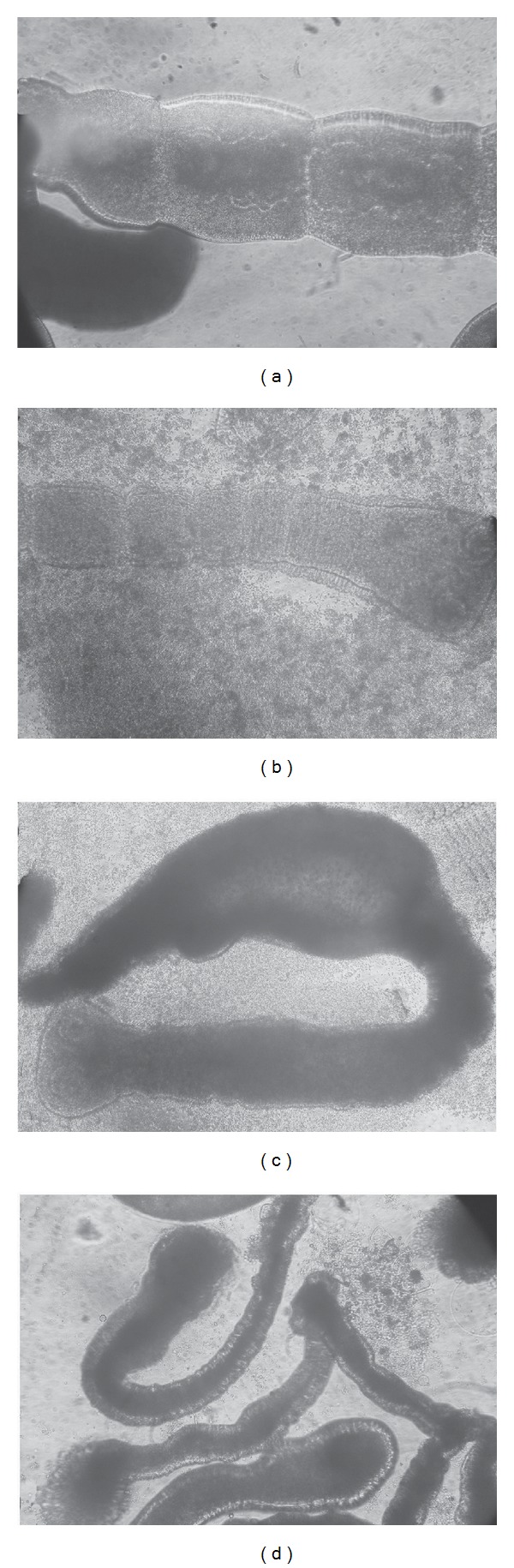
Inverted optical microscope of* Mesocestoides corti* adult worms incubated* in vitro* with thymol. (a) Control adult worm (100x). Partial view of a strobila. (b) Altered adult worm after 10 min of incubation with thymol (250 *μ*g/mL). The tegument is altered and debris of tegument could be observed in the culture medium (100x). (c) Adult worm (10 min, 200 *μ*g/mL). Note the tegumental damage and the influx of culture medium into the worm (100x). (d) Adult worms after 30 min of incubation with thymol (150 *μ*g/mL). Note the tegumental alterations with the presence of blebs and loss of morphology (100x).

**Figure 4:**
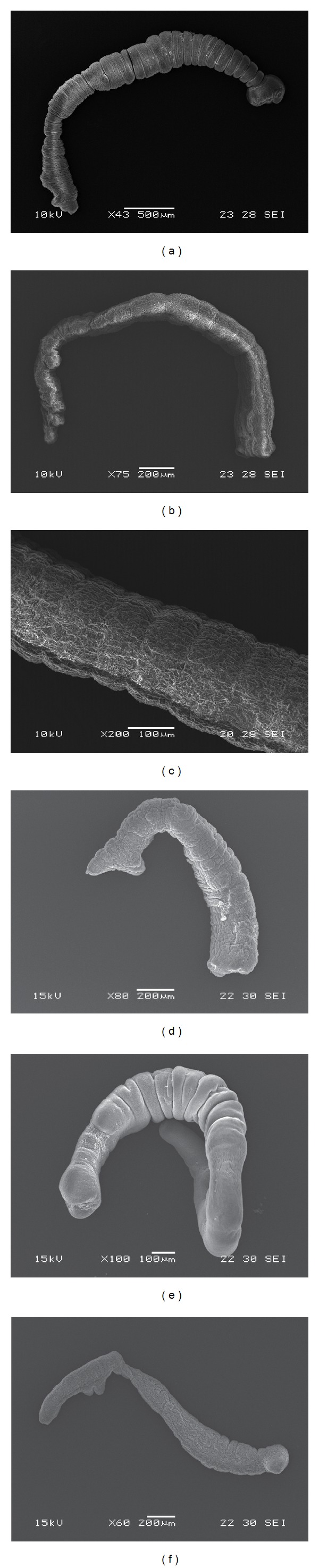
Scanning electron microscopy of* Mesocestoides corti* adult worms incubated* in vitro* with thymol. (a) Control adult worm (43x). (b) Adult worm after 15 min of incubation (250 *μ*g/mL). Note the tegumental alterations (75x). (c) Partial view of a strobila. Observe the extensive erosion of the tegument (1 h, 200 *μ*g/mL, 200x). (d) Altered adult worm (1 h, 250 *μ*g/mL). The constrictions between proglottids became difficult to distinguish (80x). (e) Adult worm after 1 h following incubation. Partial loss of microtriches (1 h, 150 *μ*g/mL, 100x). (f) Adult worm totally altered after overnight incubation (150 *μ*g/mL, 60x).

No changes in structure or ultrastructure were observed on control worms throughout the experimental period (Figures 3(a) and 4(a)). Moreover, the motility was not affected with the presence of the usual contraction movements of the body. Following a short incubation time (2–5 min) at the studied concentrations of thymol, a decrease in activity of the parasites was observed. After 30 min, a complete paralysis was noted with the higher concentrations of drug (200 and 250 *μ*g/mL). At 150 *μ*g/mL, complete paralysis was detected after 2 h following incubation.

Changes in motility coincide with the tissue damage observed at the structural and ultrastructural levels. The primary site of damage was the tegument of the parasite. After 10 min following incubation at 250 and 200 *μ*g/mL, tegumental alterations could be observed by inverted microscope alongside debris of tegument in the culture medium (Figure 3(b)). The surface of the body was extensively damaged and the presence of blebs was evident. Some worms showed damage to the posterior part of the body, which probably resulted in a total disruption of the tegumental layers and an influx of culture medium into the worm (Figure 3(c)). The same lesions in the tegument were detected after 30 min following incubation at 150 *μ*g/mL (Figure 3(d)).

Studies by SEM revealed that ultrastructural damage was produced in thymol-treated worms. After 15 min following incubation, marked tegumental alterations and the complete loss of microtriches were detected at 200 and 250 *μ*g/mL (Figure 4(b)). When segmented forms were incubated with the same concentrations of thymol for 1 h, more pronounced changes, such as loss or morphology and extensive erosion of the tegument, were induced (Figure 4(c)). Moreover, the constrictions between proglottids became difficult to distinguish or differentiate (Figure 4(d)). These specimens were considered to be dead. As it was mentioned for optical observations, at 150 *μ*g/mL changes produced by the drug treatment were detected later. After 1 h following incubation tegumental damage and partial loss of microtriches were observed (Figure 4(e)). After overnight incubation, worms were totally altered, with complete loss of morphology (Figure 4(f)).

## 4. Discussion

Development of new efficient drugs for the treatment of human and animal infections caused by cestodes is an urgent issue for pharmacologists. Over the past ten years, the main research goal in our laboratory has been the experimental chemotherapy of cystic echinococcosis. We evaluated the* in vitro* and* in vivo* anthelmintic effects of different synthetic and natural drugs [13–15, 17–20].

As opposed to larval stage of* E. granulosus*, the infection in the definitive host has not been so widely studied and comparatively fewer experimental data have been gathered [21]. Up to now research on* in vitro* cultures of adults has proven difficult, only reaching some degree of maturation in the diphasic medium [22]. For this reason, we thought that* M. corti* adult worms could be an interesting* in vitro* model for the screening of new drugs against canine echinococcosis.

No previous publications were found about the anthelmintic* in vitro* effect of thymol on cultured* M. corti* tetrathyridia and adult worms. Besides, this work is the first report of the effect of a component of essential oils on this parasite.

Tetrathyridia exposed to different concentrations of thymol showed a concentration and time-dependent effect involving morphological damage. The employment of SEM allowed us to examine, at an ultrastructural level, the effects induced by thymol on* M. corti* tetrathyridia. The main change observed after exposure was mainly in morphology, with larvae exhibiting an elongation of the body. Additionally, the presence of blebs and holes or depressions could be observed. At lower concentrations, microtriches remained unaltered. Increasing the concentration from 10 to 100 *μ*g/mL did not result in a proportional increase in the observable damage. When tetrathyridia were exposed to 250, 200, and 150 *μ*g/mL of thymol, there were increased surface alterations and damage to the larvae. The body appeared elongated and flattened, and a complete loss of morphology and microtriches was observed. The alteration of microtriches probably interferes with tetrathyridia nutrition since microtriches are directly associated with the nutrients absorption. These ultrastructural changes have also been observed on tetrathyridia cultured in the presence of free and liposomized praziquantel [5]. Furthermore, Hrčková et al. [5] observed an increase in motility of tetrathyridia. In contrast, a decrease in activity and paralysis was observed when larvae were incubated with thymol. Moreover, as evidenced in our experiments, thymol was able to kill* M. corti* tetrathyridia, since following inoculation of treated parasites in mice no parasites could be recovered after two months following inoculation in all mice infected with thymol-treated parasites.

On the other hand, the efficacy of thymol was also demonstrated* in vitro* on* M. corti* adult worms. As occurred for tetrathyridia, the effect was dose and time dependent. A correlation between the intensity of damage and the concentration of thymol was observed. Our results are consistent with those reported by Chavasse et al. [23], where adult* Schistosoma mansoni*, incubated with praziquantel, showed a decrease in activity and paralysis. Changes in motility coincide with the tissue damage observed at the structural and ultrastructural levels. The primary site of damage was the tegument of the parasite. SEM studies revealed that, even after a short incubation time, marked tegumental alterations and the complete loss of microtriches were detected.

Thymol caused severe damage to the two developmental stages analyzed. However, damage was more significant in fully segmented worms. This difference was also observed by Markoski et al. [2] working with praziquantel and albendazole. They stated that the observed effects are probably representative of those happening to intestine living adult cestode worms upon host oral treatment with these drugs.

We consider that a possible limitation of this study is the lack of* in vivo* studies. Moreover, exhaustive evaluation of thymol chemotherapeutic efficiency* in vitro* on tetrathyridia and adult worms should be undertaken.

In conclusion, the data obtained clearly demonstrated that the* in vitro* treatment with thymol is effective against* M. corti* tetrathyridia and adult worms. The results obtained on tetrathyridia are consistent with our previous observations working* in vitro* with* E. granulosus* larval forms [13]. As far as we know, this is the first time that the anthelmintic effect of a compound of a phytotherapic drug upon adult worms of cestodes is assessed. In the next step, we will investigate the* in vitro* and* in vivo* efficacy of thymol against* E. granulosus* adults.
